# Can vocal conditioning trigger a semiotic ratchet in marmosets?

**DOI:** 10.3389/fpsyg.2015.01519

**Published:** 2015-10-07

**Authors:** Hjalmar K. Turesson, Sidarta Ribeiro

**Affiliations:** Brain Institute, Federal University of Rio Grande do Norte, Natal, Brazil

**Keywords:** vocal learning, conditioning, operant, marmoset, semiotics, language disorders

## Abstract

The complexity of human communication has often been taken as evidence that our language reflects a true evolutionary leap, bearing little resemblance to any other animal communication system. The putative uniqueness of the human language poses serious evolutionary and ethological challenges to a rational explanation of human communication. Here we review ethological, anatomical, molecular, and computational results across several species to set boundaries for these challenges. Results from animal behavior, cognitive psychology, neurobiology, and semiotics indicate that human language shares multiple features with other primate communication systems, such as specialized brain circuits for sensorimotor processing, the capability for indexical (pointing) and symbolic (referential) signaling, the importance of shared intentionality for associative learning, affective conditioning and parental scaffolding of vocal production. The most substantial differences lie in the higher human capacity for symbolic compositionality, fast vertical transmission of new symbols across generations, and irreversible accumulation of novel adaptive behaviors (cultural ratchet). We hypothesize that increasingly-complex vocal conditioning of an appropriate animal model may be sufficient to trigger a semiotic ratchet, evidenced by progressive sign complexification, as spontaneous contact calls become indexes, then symbols and finally arguments (strings of symbols). To test this hypothesis, we outline a series of conditioning experiments in the common marmoset (*Callithrix jacchus*). The experiments are designed to probe the limits of vocal communication in a prosocial, highly vocal primate 35 million years far from the human lineage, so as to shed light on the mechanisms of semiotic complexification and cultural transmission, and serve as a naturalistic behavioral setting for the investigation of language disorders.

## Introduction

Since Darwin’s (1872) comparative study of emotions, psychological continuity across species has been a dominant view in biology. However, the complexity of human communication has often been taken as evidence that our language reflects a true evolutionary leap, bearing little resemblance to any other animal communication system (Tomasello and Call, 1997; Tomasello, 1999). Scientists have argued bitterly over whether animals possess language or not (Marler, 1970; Griffin, 1984; Savage-Rumbaugh, 1990; Pepperberg et al., 1997; Gelman and Gallistel, 2004; Tomasello et al., 2005). Throughout most of the past century, the putative uniqueness of our language posed serious evolutionary and ethological challenges to a rational explanation of human communication. How did the phonology, syntax and semantics of human language evolve if apes, our closest relatives, appear to be so laconic?

Yet, the increasingly better sampling of animal behavior in the past decades showed that apes and other primates possess vocal referential communication (e.g., Seyfarth et al., 1980), cultural transmission (e.g., Whiten et al., 1999), and even simple combinatorial syntax (e.g., Ouattara et al., 2009). We actually share with certain mammalian and avian species the fundamental sensorimotor mechanisms required for the perceptual and motor aspects of vocal learning (Petkov and Jarvis, 2012), such as the expression in cortico-striatal and cortico-cerebellar circuits of transcription factor FOXP2, which regulates the normal development of human speech (Konopka et al., 2009; Scharff and Petri, 2011). A comprehensive computational analysis of gene expression profiles measured in various brain regions recently allowed for the identification of several functionally and molecularly analogous brain regions for birdsong and human speech (Pfenning et al., 2014). Nevertheless, it remains unclear why have other animals failed to evolve the higher traits of human language.

To shed light on this problem, we begin by reviewing the contributions of semiotics to the problem of vocal communication. Next we review the empirical literature on vocal learning across species, with a focus on the comparison between New and Old World primates. Finally, we present a series of ongoing experiments aimed at probing the semiotic limits of vocal communication in marmosets.

## Meaning by Resemblance: Iconic Representations

Semiotics is the logical system developed by Peirce (1958, 1998) to formally describe the communication of an object to an interpretant by way of a sign. In this system, the relationship between sign and object strictly derives from three—and only three—different kinds of mental processes for the establishment of meaning: icon, index or symbol.

If a sign resembles the physical properties of an object, it is said to be an icon of this object. If a sign has spatio-temporal contiguity with an object, it is said to be an index of this object. If a sign represents an object by an entirely arbitrary rule or convention established among interpreters, it is said to be a symbol of that object (Peirce, 1958, 1998). A critical difference between an index and a symbol is that the former requires the simultaneous presence of the object, while the latter often occurs in the complete absence of the object. No other categories exist beyond these three, and by definition, a sign must belong to at least one of them.

Icons are the simplest form of sign, because they convey meaning through the sheer physical similarity with the object, without the need of further abstraction. Arguably, iconic communication would not be simple and perhaps not even possible if parts of the brain did not represent perceptual information in an iconic manner (Ribeiro, 2010). Visual, auditory and somatosensory inputs ascend from the periphery to the central nervous system in a quite organized manner, leading to orderly topographic maps in primary sensory thalamus and neocortex for positions in physical space (Hubel and Wiesel, 1959), sound frequencies in acoustic space (Hind, 1953), and locations along the body surface (Mountcastle, 1957; Mountcastle et al., 1957; Simons and Woolsey, 1979). As a consequence, object representations in these early processing areas are largely congruent with the objects represented. A well-known example of this feature was provided by the use of 2-deoxyglucose uptake by stimulated neural tissue to imprint a visual grid on the primary visual cortex of macaque monkeys (Tootell et al., 1982). While the experiment revealed the high degree of isomorphism between stimulus and response, it also made explicit that this response is modified by the cortical magnification factor that over-represents the center of the visual field in detriment of the periphery.

Another telling example of the interplay between iconic representations and hardwired filters for ecologically-relevant stimuli can be found in the canary, a seasonal songbird with a characteristic syllable repertoire. The caudomedial nidopallium (NCM) of the canary, an auditory region homologous to the mammalian primary auditory cortex, responds to natural canary whistles according to their frequencies, with low-pitched whistles mapping dorsally in NCM, and gradually more ventral mapping as the pitch increases (Ribeiro et al., 1998). Yet NCM cannot be said to be strictly tonotopic, because artificial stimuli with the same fundamental frequency fail to produce a well-localized response in NCM: computer generated tones activate more diffuse groups of neurons, and guitar notes stripped of their harmonics even more so, without any clear topography. Thus, NCM is “whistletopic” rather than tonotopic, i.e., it carries an orderly representation of the environment that is clearly tuned to the acoustic features of natural stimuli.

If iconic representations in primary cortical areas are more the rule than the exception, examples of iconic communication among non-human animals are not compelling. Inter-specific communication by sheer vocal imitation (Owen-Ashley et al., 2002) is rare; for instance, to date there is no evidence that animals produce onomatopoeic alarm calls to warn against predators. Still, the notion that onomatopoeias played an important role in the early evolution of words is tempting, with old roots in philosophy and linguistics (von Humboldt, 1836; Jakobson and Waugh, 1979; Hinton et al., 1994; Magnus, 2010), and fresh support from studies of infant psychology (Maurer et al., 2006; Imai et al., 2008; Kita et al., 2010) and neuroscience (Osaka et al., 2004; Aziz-Zadeh et al., 2006; Garcia et al., 2014). Judgment of this issue is tied to the relatively recent advent of computer-aided devices to investigate spontaneous behavior of other species in the field. As more studies with better recording techniques are performed, discoveries regarding the natural use of iconic communication may still lead to substantially different conclusions.

## Meaning by Spatio-Temporal Contiguity: To Point, to Look, to Indicate

In comparison with icons, indexes constitute a much more flexible type of sign, since the same pointer can be used to mean an endless variety of different objects. The highly informative act of indicating with the finger or gaze is so ubiquitous across different human cultures that one must conclude that its origins are ancient in the *Homo* lineage. Indeed, indexes are far from being exclusive of human communication. A number of studies suggest that chimps are capable of taking conspecifics gaze direction into account (Call et al., 1998; Hare et al., 2000, 2001). Apes can also follow human pointing, given appropriate methodologies and/or human exposure (Mulcahy and Call, 2009; Lyn et al., 2010; Leavens et al., 2012; Hopkins et al., 2013). Rhesus monkeys follow both human pointing and gaze (Hauser et al., 2007), and even corvids have been shown to follow gaze (Bugnyar et al., 2004; Bugnyar, 2011). The fact that human pointing is followed by domesticated dogs and foxes (Hare et al., 1998, 2002, 2005; Hare and Tomasello, 1999), as well as trained dolphins (Pack and Herman, 2004), suggests that indexical learning can be greatly boosted within a few generations by trait selection, and even within a single generation, by learning from interactions with point users (e.g., human trainers). Altogether, these studies indicate that the capacity for index-based communication is widespread and therefore not a suitable candidate to explain the uniqueness of human language. But it may well be that quantitative differences in the ability to use indexes (Goldin-Meadow, 2007), supported by a large repertoire of arbitrary vocal calls (Ghazanfar et al., 2007), allowed our hominid ancestors to initiate the cultural ratchet that led to contemporary human language.

Indexes are very useful to signal to other individuals the presence of conspecifics, predators, preys, or feeding opportunities, and therefore a likely preadaptation of indexical communication is prosociality, i.e., the ability to display behaviors that favor other individuals even in the absence of immediate self-benefit. Prosociality is a widely distributed trait among mammals, from rats (Daniel, 1942; Ben-Ami Bartal, 2011; Márquez et al., 2015) to apes (Horner et al., 2011; von Rohr et al., 2012; Hobaiter and Byrne, 2014; Hobaiter et al., 2014).

Yet, as prosocial as apes can be, they seem to have little capacity for using indexes to share intentions on the execution of collaborative activities (Warneken et al., 2006; but see Leavens et al., 2005). Several researchers have proposed that the key difference between humans and non-humans is shared intentionality, a cornerstone of our exquisite ability to teach and learn, able to create a cultural ratchet that leads to the fast transmission of any useful cultural innovation (reviewed in Tomasello et al., 2005). Joint attention in humans begins at 9–12 months with gaze following, the use of adults for social reference, and the imitation of adult gestures (Bakeman and Adamson, 1984; Moore and Dunham, 1995). The understanding that there are other minds begins as babies start to recognize voluntary behaviors with overt or covert goals (Meltzoff, 1988; Carpenter et al., 1995, 1998). As children develop, adults become outside entities whose attention can be called, both as observers of the child’s actions and as producers of behaviors desired by the child. Lack of shared intentionality and deficits in index usage seem to be an important component in autism (Tomasello et al., 2005).

Point following and the partaking of goals appears to be a bottleneck for the learning of indexical signs, and for this reason we designed experiments to quantitatively assess the conditions that allow shared intentionality to arise between pairs of independent vocal agents, either overtly or covertly. These experiments are detailed below, but before they are properly introduced it is necessary to present the quite controversial topic of symbolic communication.

## Meaning by Convention: Are We the Symbolic Species?

The search for the key difference between human language and the communication systems in other species led to the proposal that the use of symbols is the distinctive property that sets us apart from the other animals (Deacon, 1998). According to this view, non-human animals are limited to iconic and indexical communication, and therefore utterly incapable of using symbols, the most powerful and flexible sign type. Supposedly the “symbolic species hypothesis” is grounded on the Peircean semiotics, but a closer inspection of semiotics shows that the definition of symbol entirely matches the empirical evidence of referential communication observed in different animal species.

Chimpanzees in captivity can learn to use man-made lexigrams to refer to dozens of different objects and actions (Savage-Rumbaugh et al., 1986; Gillespie-Lynch et al., 2014), effectively expanding their ability to communicate with caregivers and experimenters. However, it has been argued that lexigram use by chimpanzees does not represent true symbolic communication, but rather functional communication based on the instrumental learning of the specific contingencies of the experimental setting (Seidenberg and Petitto, 1987).

Field studies of spontaneous animal communication effectively bypass this concern. Adult vervet monkeys (*Cercopithecus aethiops*), Old World primates from the African savannahs, naturally display three kinds of alarm calls that correspond specifically to the presence of terrestrial, aerial or slithering predators. Upon hearing alarm calls uttered by an adult, other adult monkeys will promptly react to protect themselves, hiding above or below trees in the case of aerial or terrestrial predators, respectively, or moving aside and scanning the ground in the case of snakes. Juvenile vervet monkeys are able to emit the same vocalizations but do so out of context, and therefore do not produce escape reactions in the adults.

Field observation of vervet monkey behavior argues strongly for the symbolic quality of alarm calls among adults (Struhsaker, 1967; Seyfarth et al., 1980). First, the proper context of use of these calls is slowly learned by repeated pairing of alarm call (auditory stimulus) and predator (visual and/or olfactory stimulus), denoting the gradual establishment of a social convention regarding the interpretation of these alarms. Second, experimental playbacks of these alarm calls produce proper escape reactions in the absence of any predator among adults, exemplifying a trademark of symbolic communication, namely that meaning is conveyed in the absence of the object (Seyfarth and Cheney, 1988). Comparable communication systems have been documented in close relatives, Campbell’s monkeys (*Cercopithecus campbelli*) and Diana monkeys (*Cercopithecus diana*; Zuberbühler, 2000, 2001). More recently, field research of chimpanzees revealed the use of intentional calls to warn against a predator model (Schel et al., 2013).

Computational simulations of the interactions of artificial creatures representing vocalizing preys and their predators suggest that the referential code that ascribes specific meaning to each call type arises by chance, through random variations that get to be established and maintained over time. This occurs when the prey-predatory ratio is sufficiently large for the prey population to survive long enough for the establishment and spread of the code (Queiroz and Ribeiro, 2002; Gudwin et al., 2003; Loula et al., 2004; Ribeiro et al., 2007).

Since the original findings in vervet monkeys, the occurrence of referential communication regarding specific predator types (Macedonia and Evans, 1993) has been demonstrated in a variety of non-primate species, including ground squirrels (*Spermophilus* spp.; Owings and Hennessy, 1984), chickens (*Gallus gallus domesticus*; Gyger et al., 1987; Evans and Evans, 1999), prairie dogs (*Cynomys gunnisoni*; Slobodchikoff et al., 1991; Kiriazis and Slobodchikoff, 2006), tree squirrels (*Tamiasciurus hudsonicus* Greene and Meagher, 1998), dwarf mongooses (*Helogale undulata*; Beynon and Rasa, 1989), suricates (*Suricata suricatta*; Manser, 2001; Manser et al., 2001). Similarly, bottlenosed dolphins have been reported to understand human gestures as symbolic representations of body parts (Herman et al., 2001).

Altogether, the empirical and computational results of referential communication among a wide variety of species clearly contend against the notion that humans are the only species to employ symbols. Rather, referential communication in non-human species conform to the notion of dicent symbol in Peircean semiotics, i.e., a symbol that functions “like an index” because its object is “a general interpreted as an existent” (Peirce, 1998). In the semiotic framework, what distinguishes human language from the communication system of other species is our ability to concatenate symbols of symbols of symbols, namely what Peirce labeled “argument.”

## How Informative is the Structure of Arguments?

Several animal species use sequences of vocalizations to communicate. Notably, birdsong is typically composed of a sequence of phrases, each comprising a string of repeated syllables (Williams and Staples, 1992). In many species, adult males display a stable sequence of vocalizations, with little variation from bout to bout within a season (e.g., canary: Nottebohm et al., 1981) or even across seasons (e.g., zebra-finch: Walton et al., 2012), especially when singing toward a female (Sossinka and Bohner, 1980).

In extreme cases, like in the nightingale, the huge size of the syllabic repertoire leads to a very flexible display (Hultsch et al., 2004). Likewise, mockingbirds have the ability to mimic the vocalizations of other species, resulting in very complex sequences akin to improvisation (Derrickson, 1987). Nevertheless, little or no meaning seems to be ascribed to phrase order, with the clear exception of introductory notes (Price, 1979), which may indicate whether animals are singing directly to a conspecific, or singing alone (Jarvis et al., 1998).

There is no indication that songbirds can shuffle phrases to generate a combinatorial reference code. In fact, this ability seems to be exceedingly rare, uniquely human if not for the (rather limited) example of compositionality in some Old-World primates, such as the putty-nosed monkey (*Cercopithecus nictitans*), which can employ two vocalizations in a combinatorial manner (Arnold and Zuberbühler, 2006). With the sole exception of suffix usage among Cercopithecines (Ouattara et al., 2009; Coye et al., 2015), and human-tutored apes (Savage-Rumbaugh et al., 1986) and dolphins (Richards et al., 1984), symbolic arguments seem to be exclusively humans.

The notion that the structure of arguments carries important information is tightly linked to graph theory, which deals with networks of interconnected elements, such as successive words during a conversation. Graph theory was initiated by Leonhard Euler in the eighteenth century, and was greatly developed in the twentieth century (Bollobás, 1998) with ever-expanding applications in physics, chemistry, biology and indeed any field in which networks play a key role (Milo et al., 2002; Sporns, 2010). In the context of vocal communication, a graph represents the temporal sequence of vocalizations, with each vocalization represented as a node, and the transition between consecutive vocalizations represented as a directed edge (Ferrer I Cancho and Solé, 2001). The overarching applicability of graph theory to communication suggests that it is particularly well suited to causally bridge very different levels of description of the phenomenon, such as systems neurophysiology and linguistics. If activity within an interconnected network of neurons is very aptly described as a directed graph (Changeux, 1997), so is the sequential production of utterances that characterize vocal communication in general, or human language in particular.

**FIGURE 1:**
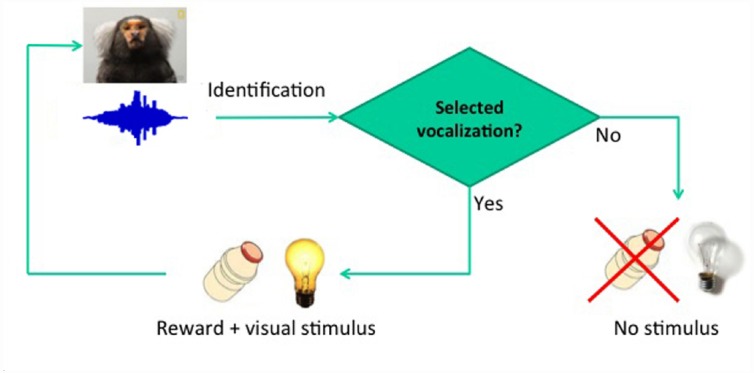
**Basic conditioning paradigm.** The delay between vocalization and reward was fixed at 2 s.

**FIGURE 2:**
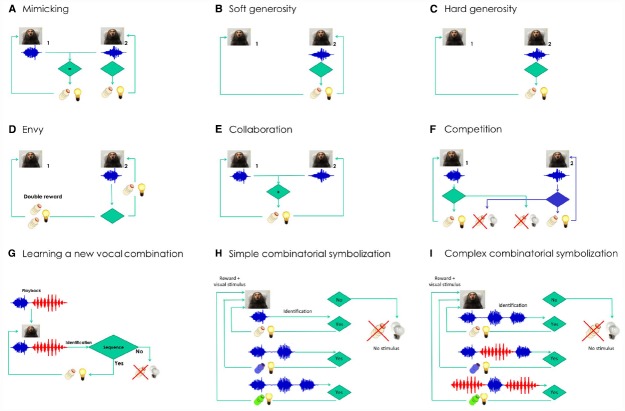
**Semiotic experiments using conditioned marmoset vocalizations.** Does social identity matter for each of the experiments? Learning curves are expected to be steeper and shorter when the interacting animals are from the same family. **(A)** Mimicking experiment: Marmoset 2 will be rewarded only when able to deliver the same kind of vocalization produced by marmoset 1 (e.g., *phee*, in blue). **(B)** Soft generosity experiment: Marmoset 1 will be rewarded when marmoset 2 is rewarded after a correct vocalization. **(C)** Hard generosity experiment: When marmoset 2 produces the correct vocalization, only marmoset 1 will be rewarded. **(D)** Envy experiment: Marmoset 1 will get double reward when marmoset 2 is rewarded after a correct vocalization. **(E)** Collaboration experiment: The two marmosets will be rewarded only when able to deliver the same kind of vocalization in close temporal proximity. Can the task be built with several animals in tandem? **(F)** Competition experiment: A properly-timed vocalization by marmoset 2 will allow it to steal the reward from marmoset 1, and *vice versa*. How many turn-taking will be reached as a function of kinship and satiation? **(G)** Learning a new combination of vocalizations experiment: The marmoset will be rewarded if it manages to reproduce the artificial sequence that will be played back, composed of two different vocalizations (*phee* + *twitter*, in red). **(H)** Simple combinatorial symbolization. The experiment involves learning to produce a different number of *phee* pulses to obtain rewards of different appetitive value. Shifts in the distribution of *phee* pulses will be expected depending on the contingency established. **(I)** Complex combinatorial symbolization. The experiment involves learning to produce different combinations of call types to obtain rewards of different appetitive value (in this example, *phees* and *twitters*). Shifts in the distribution of call sequences will be expected depending on the contingency established.

The usefulness of structural speech features to discriminate pathological and non-pathological reports has recently been demonstrated. Specific graph attributes allow the quantitative discrimination of schizophrenic versus manic subjects, even when non-psychotic subjects are included in the sample (Mota et al., 2012, 2014). Similar analyses successfully discriminate patients with Alzheimer’s disease from patients with mild cognitive impairment (Bertola et al., 2014). Overall the results indicate that the graph-theoretical analysis of vocalizations is key to the study of language-related deficits in humans, and may have major applicability in animal models of diseases such as autism, such as the marmoset (Shen, 2013). Effective screening for phenotypes of interest may be provided by semiotic experiments based on conditioning (Figure 1), designed to track essential quantitative differences in the structure of vocalizations as they become indexes, then dicent symbols, and possibly arguments (Figure 2). Before proceeding to the explanation of these experiments and to the justification of the choice of marmosets, it is important to review the known boundaries of vocal learning in primates.

## Vocal Flexibility in Non-Human Primates

For symbolic communication to be powerful (i.e., communicate a great number of objects), and/or flexible (i.e., communicate new objects), two aspects must concur. First, the ability to learn to interpret new symbols. Second, the ability to use and produce new symbols for adaptive operation within a given context. While there is plenty of evidence of the former (Savage-Rumbaugh et al., 1978; Savage-Rumbaugh and Romsky, 1989; Gelman and Gallistel, 2004), examples of the latter are substantially more limited among non-human primates. Below we briefly review the findings on primate vocal flexibility, understood as the adaptive, cognitively mediated control over vocal production, comprising usage learning, production learning, and acoustic modifications in the presence of noise (Seyfarth and Cheney, 2010).

## Vocal Production Learning in Non-Human Primates

Vocal production learning refers to the ability to learn to produce a new vocalization. This can be a modification of a vocalization already in the animal’s repertoire, or the production of an entirely new vocalization. Historically, the focus has been on entirely new vocalizations since it is hard to ensure that what appears to be a modified vocalization is not merely a preexisting vocalization that simply was not experimentally observed before (and thus only a matter of usage learning; Janik and Slater, 1997, 2000; Tyack, 2008). Among mammals, imitation of completely novel, often anthropogenic, sounds has only been reported for dolphins, elephants, harbor seals, and humans (Tyack, 2008). However, this narrow way of studying vocal production learning inadvertently excludes many species that at a closer look display modifications of vocalizations beyond the solely motivational or developmental.

One such (subtle) type of vocal modification is the convergence of calls, which takes place when some acoustic features of different individuals’ calls converge, often as new social groups form. This has been reported in several species, and among them, primates. In two influential studies Snowdon and Elowson (1999) demonstrated that certain acoustic features of the trill calls produced by pygmy marmosets (*Cebuella pygmaea*)—an intragroup communication call, apparently used for maintaining group cohesion (Snowdon and Hodun, 1981)—converged after different groups were placed in a common acoustic environment (Elowson and Snowdon, 1994), as well as when individuals were paired (Snowdon and Elowson, 1999). This suggests that pygmy marmosets indeed have some capacity for learning their vocal production, expressed in response to changes in social environments. Similar findings have followed, demonstrating for example the convergence of food grunts after the merging of two chimpanzee groups (Watson et al., 2015), and the convergence of pant hoots (a long distance call) to a version shared within the group (Marshall et al., 1999).

Thus, it seems that non-human primates might be capable of vocal production learning, and thus have more vocal flexibility than what was apparent when using the ability to imitate novel vocalizations as a strict requirement (Elowson and Snowdon, 1994; Marshall et al., 1999; Snowdon and Elowson, 1999; Watson et al., 2015). However, the above-mentioned acoustic modifications are subtle, and possibly caused by other factors than learning. Thus, the hard evidence has to be carefully examined, and followed up by better-controlled studies able to rule out the possible confounds. The next section presents some of these confounds.

## Vocal Usage in Non-Human Primates

Learning in which context to produce a call is often labeled vocal usage learning and it demonstrates a degree of cognitive control over vocalizations. This kind of vocal flexibility is generally studied with conditioning experiments in which the animal is rewarded for producing calls (any call or a particular type of call). Several studies have presented results suggesting that non-human primates can be conditioned to produce at least some types of vocalizations in response to particular contexts (Sutton et al., 1973; Pierce, 1985; Hihara et al., 2003; Hage et al., 2013), while others have reported failures (Yamaguchi and Myers, 1972; Aitken and Wilson, 1979).

A classic and often cited study offered food reward to three juvenile rhesus monkeys (*Macaca mulatta*) for producing more and longer calls (Sutton et al., 1973). After 3–4 weeks of training, two of the subjects increased their average number of calls per session, and all three showed an increase in call duration. Pierce (1985) reviewed 12 early attempts to condition the vocalizations of non-human primates and came to the conclusion that they indeed have a considerable amount of control over their own vocal output. However, as in the study from Sutton et al. (1973), several of the results mentioned by Pierce (1985) could possibly be caused by motivational factors instead of learned vocal control.

## Criticism: Observational Studies

A major problem with field studies is that they are purely observational, and thus allow for a whole slew of confounding variables to affect the results. For example Arcadi (1996) and Mitani et al. (1999) showed that acoustic features of the pant-hoot in chimpanzees varies between geographically separated populations. This variation has been interpreted by other authors as an indication that vocal development in chimpanzees involves learning (e.g., Arcadi, 1996). However, careful analysis of a number of environmental factors suggests that the acoustic difference between calls from the two groups does not have to be an effect of learning. For example, the two groups lived in different habitats with different acoustics due to varying density and type of forestation (i.e., primary versus secondary forest), and thus the observed differences in the calls could instead reflect adaptations to different acoustic environments. Further, the amount of interfering noise the pant hoots were subjected to differed according to varying levels of biodiversity in the two habitats. Also the average body size likely differed between the two groups, with corresponding changes in the acoustic features of the calls that depend on body size. Finally, the two groups were separated by such a large geographical distance that between-group genetic differences could not be excluded (Mitani et al., 1999). Thus, what superficially seemed to imply some form of vocal learning, might only be a consequence of environmental factors.

## Criticism: Motivational Factors

A recurrent problem in many of the studies, both on vocal production learning, and vocal usage, is the influence of the motivational or arousal state (Owren et al., 2011; Hage et al., 2013). A number of acoustic features vary with arousal level. For example, call rate, fundamental frequency and call duration all go up with increased arousal (Rendall, 2003). The level of arousal, in turn, can be influenced by a number of factors, including changes in social context and food (Braesicke et al., 2005; De Marco et al., 2011; Machado et al., 2011). Thus, when the animal’s context changes this can induce changes in arousal level, and consequently also in some acoustic properties of the calls. To avoid this, utmost care needs be taken to dissociate motivational factors from experimental manipulations and measures.

To date, this has not been systematically done. For example, Sutton et al. (1973) demonstrated that the duration of the *coo* call in rhesus monkeys increased during a conditioning experiment. The *coo* call is produced in multiple contexts, and among them when food is available (Hauser and Marler, 1993). Since call duration increases with arousal, and arousal can increase in the presence of food, it is not unlikely that what appears as the subjects learning to produce longer calls for reward is merely a matter of the subjects learning to associate a particular context with food, and that this increases arousal. Similar alternative explanations can be leveraged to most published studies (see Owren et al., 2011; Hage et al., 2013, for a list of several of these studies).

At present, the most convincing study on vocal conditioning in non-human primates comes from Hage et al. (2013). They trained two rhesus monkeys to produce vocalizations for reward in the presence of a visual cue. One of the two subjects was further trained to produce two different vocalizations, coo and grunt. Which of the two calls was rewarded in a particular trial was indicated by two distinct visual cues. The subject learned to do this and, within the same experimental session, produced nearly exclusively coo calls when the cue indicated so, and grunts when that was indicated. Very few calls were produced in the wrong context. Even though both calls can be considered to be food associated (Hauser and Marler, 1993; Owren and Casale, 1994)—and thus to reflect arousal—the amount and type of reward was equal in both conditions. This means that food-related arousal should also be equal across conditions, and thus it cannot explain the differential calling depending on the visual cue presented. Hage et al. (2013) represent the only clear exception in the literature, which is otherwise confounded by arousal effects.

In summary, in face of the numerous books and review articles written on the evolutionary history of human speech in general and vocal flexibility in non-human primates in particular, it is surprising that better-controlled methods for characterizing the limits of vocal learning in non-human animals have not been developed. Nearly all the positive evidence lack appropriate controls to rule out confounding effects such as differences in body weight, habitat, and most importantly motivation. Future studies must be designed to effectively dissociate motivational effects from the observable changes in vocal usage and production. The best would be to show that the vocal modifications could be driven in two opposite directions by equally arousing context/social environments. In the next section we present some key methodological improvements in this regard.

## If a Marmoset Could Speak, We Should Strive to Understand

A large body of evidence indicates that human language shares many features with communication in other animals, and that the greatest differences lie in the superior human capacity for symbolic compositionality, fast vertical transmission of new symbols, and irreversible accumulation of novel adaptive behaviors that characterizes a cultural ratchet. We hypothesize that increasingly-complex vocal conditioning of an appropriate animal model may be sufficient to trigger a semiotic ratchet, evidenced by progressive sign complexification, as spontaneous contact calls become indexes, then symbols, and finally arguments.

An adequate animal model for testing this hypothesis should be amenable to laboratory research, have a rich vocal repertoire, and show prosocial behavior characterized by cooperative signaling and parental scaffolding. There is a continuum across primates regarding the importance of posterior–anterior cortical interactions for the perception and production of vocalizations, by way of the arcuate fasciculus (Rilling et al., 2008). Regions homologous to the Wernicke area for speech perception have been identified in chimpanzees (Gannon et al., 1998), while regions homologous to the Broca area for orofacial control and speech production have been recognized even in the common marmoset (*Callithrix jacchus*; Miller et al., 2010; Simões et al., 2010) a highly vocal New World monkey species split from Old World monkeys around 40 million years ago (Worley et al., 2014).

Marmosets are cooperative breeders (Koenig, 1995), prosocial (Burkart et al., 2007; Burkart and van Schaik, 2013), and capable of attributing intentions to conspecifics (Burkart et al., 2012). Similarly to the development of human speech (Goldstein and Schwade, 2008), the maturation of vocal communication in marmosets depends on parental scaffolding, in the form of contingent vocal (social) feedback that seems to shape the transition to adult vocalizations (Takahashi et al., 2013, 2015). These findings point to marmosets as an adequate animal model for the investigation of the development of indexical, symbolic, and argumental communication.

Below we outline a series of conditioning experiments designed to test the semiotic ratchet hypothesis in marmosets: Is it possible to trigger a cultural ratchet by rewarding specific individual vocalizations, so as to gradually build meaning? The experiments described in Figure 2 aim to investigate the experimental and possibly natural occurrence of indexes and symbols in marmosets, based on the timing of auditory and visual events, such a gaze orientation, vocalization, and appetitive behavior. The experiments were designed to effectively dissociate motivational effects from learning-related vocal changes, are suitable for a graph-theoretical analysis of the structure of vocalization sequences, and allow for the investigation of shared intentionality within and across social groups.

The first step is vocal conditioning, using the real-time detection of specific calls from a specific individual to deliver reward (Figure 1). Conditioning will be conducted so as to effectively dissociate motivational effects from learning-related vocal changes, first rewarding animals for producing multiple pulses in tandem, and then alternating to the exclusive reward of single-pulse calls. This should lead to substantial variations in the number of pulses produced, providing a direct control for arousal effects.

We will then initiate a series of experiments that take advantage of inter-animal differences in social rank and kinship, comparing results obtained from pairs of animals within and across families. These experiments involve imitation (Figure 2A), soft and hard generosity (Figures 2B,C), envy (Figure 2D), collaboration (Figure 2E), competition (Figure 2F), and learning a new combination of vocalizations (Figure 2G). In all these experiments the role of prosociality will be assessed within and across families. These experiments propose to push the envelope of the complexity of these vocal interactions, measuring the extent of their constraint by social bonds.

We predict that calls will be at first interpreted as prospective indexes of rewards, initially surprising but very reliable, until by sheer repetition the call will transit into a dicent symbol of the reward, to be used voluntarily even in the absence of any appetitive drive (e.g., Figure 2C). Experimenting with gradually longer delays between call and reward should also favor symbolic emergence, due to the ever-increasing duration of the time spent in the absence of the object. A further step would be to explore the potential for combinatory semantics in the marmoset, by offering a variety of different rewards for either *phee* calls of different pulse-lengths (Figure 2H), or specific combinations of *phee* calls and another call type, the *twitter* (Figure 2I).

In summary, we propose that conditioning experiments can provide a fertile empirical framework for the investigation of vocal communication in non-human primates, going beyond perceptual learning to investigate two main directions, namely the emergence of symbolic competence, and the possible capacity for the production of arguments, i.e., symbolic combinatorial sequences. To test whether a semiotic cultural ratchet was indeed triggered, we will assess whether vocal conditioning becomes faster over time, with an acceleration of cultural transmission across generations. The cultural transmission of correct task execution from parents to infants even without explicit training would be a strong indication that a ratchet has been initiated. Another prediction made by the semiotic ratchet hypothesis is the absence of cultural fallbacks, i.e., once established, a new conditioned communication system should remain stable.

The experiments proposed here also have the potential to serve as a naturalistic behavioral setting for the investigation of language disorders. Structural features of pathological speech in humans, such as the reduced connectivity of the discourse among patients with schizophrenia or bipolar disorder (Mota et al., 2012, 2014), or the reduced density and diameter of speech graphs produced by patients with Alzheimer’s disease (Bertola et al., 2014), have great potential as biomarkers for a dense quantitative screening of language deficit phenotypes in transgenic marmosets (Shen, 2013). Animals with autistic phenotypes should display great difficulty in learning tasks with an important social aspect (Figures 2A–F) while possibly performing well in solo tasks (Figures 2G–I). Ultimately, understanding the processes that generate complex vocal communication in primates may prove crucial to our understanding of the evolution of human language, while at the same time shedding light on the mechanisms underlying its disorders.

### Conflict of Interest Statement

The authors declare that the research was conducted in the absence of any commercial or financial relationships that could be construed as a potential conflict of interest.
